# Trans-Spinal Direct Current Stimulation in Spasticity: A Literature Mini-Review

**DOI:** 10.3389/fstro.2022.921450

**Published:** 2022-07-01

**Authors:** Maria A. Estudillo-Guerra, Ines Mesia-Toledo, Noga Rogel, Nader Yaghoubi, Zaghloul Ahmed, Randie Black-Schaffer, Leon Morales-Quezada

**Affiliations:** ^1^Department of Physical Medicine and Rehabilitation, Harvard Medical School, Spaulding Research Institute, Spaulding Rehabilitation Hospital, Boston, MA, United States; ^2^Sackler School of Medicine, Tel Aviv University, Tel Aviv, Israel; ^3^PathMaker Neurosystems Inc., Boston, MA, United States; ^4^Department of Physical Therapy, Center for Developmental Neuroscience, College of Staten Island, Staten Island, NY, United States; ^5^Graduate Center, City University of New York, New York, NY, United States

**Keywords:** spasticity, stroke, neuromodulation, trans-spinal direct current stimulation, tsDCS

## Abstract

Spasticity is common after a stroke and has a negative impact on functional and quality-of-life measures. There is an unmet medical need to provide safe and effective treatment using non-pharmacological approaches. Trans-spinal direct current stimulation (tsDCS) is an emerging modality for non-invasive neuromodulation that induces reduction of spinal excitability leading to a decrease in spasticity. We describe current treatment options for spasticity, including a literature review about the use of tsDCS in patients with spasticity. We found four clinical studies that used tsDCS to treat spasticity for different neurological conditions including hereditary spastic paraplegia, upper extremity spasticity following stroke, multiple sclerosis, and incomplete chronic spinal cord injury. Spasticity was the primary outcome in three of the studies and a secondary outcome in the final study. The three studies that addressed spasticity as the primary outcome found that active tsDCS decreased spasticity compared to sham. These studies suggest that tsDCS can modulate spinal motor and sensory spinal pathways through the use of specific electrode montages and stimulation parameters. This therapy can improve motor functions and may represent a viable treatment option for spasticity.

## Spasticity in Stroke

Spasticity prevalence after stroke is highly variable, ranging from 17 to 43% in survivors 3 months post-stroke (Wissel et al., 2013). Up to 76% of post-stroke survivors lose motor function in the affected upper extremity (Rathore et al., 2002; Urban et al., 2010). Within the upper limbs, spasticity is most common at the elbow and the wrist, predominantly in the flexor muscles. It is often accompanied by impaired motor function, pain-associated limited range of motion, and loss of dexterity and coordination (Thibaut et al., 2013; Opheim et al., 2014). Correlations have been established between high degrees of post-stroke spasticity and significantly reduced range of motion in the upper limb (Pizzi et al., 2005). Similarly, 72% of post-stroke spastic patients have reported shoulder and elbow pain (Bender and McKenna, 2001). Overall, spasticity affects the quality of life and is highly detrimental to activities of daily living.

### Spasticity Management

The two main approaches for spasticity management include physical and pharmacological interventions (Stevenson, 2010). Spasticity is typically treated initially with less-invasive modalities and then progresses toward more invasive actions. Physical therapy represents the first line of treatment, including various strategies such as positioning, prolonged muscle stretching, splinting, motor-level stimulation, passive stretching, and range of motion exercises that may limit muscle contractures and reduce hyperactivity for a short period time. Additionally, orthoses are frequently used as a complement to physical therapy sessions. Pharmacological treatments of spasticity act on the central nervous system or directly on the muscles, and administration can be oral, intrathecal, or intramuscular injection. Some oral treatments include baclofen, tizanidine, gabapentin, dantrolene, and benzodiazepines. Baclofen, a GABA agonist, is commonly used against spasticity, though potential adverse effects including sedation, fatigue, and drowsiness limit dosing and utility, and the evidence base for its use in stroke is limited (Francisco and McGuire, 2012; Lindsay et al., 2016). The implanted intrathecal baclofen pump allows direct application of baclofen to the intrathecal space without sedative effects. While of great effect in spastic paraplegia, its use in hemiplegic stroke, is limited by its lesser effect on upper compared to lower extremity spasticity. Arousal side effects similarly limit the use of tizanidine after stroke and brain injury and increasing concern for the addictive properties of benzodiazepines has reduced their use in all spastic populations.

The botulinum neurotoxin (BoNT) is an injected agent that is used to treat focal spasticity. BoNT type A (BoNT-A) selectively inhibits acetylcholine release at the neuromuscular junction, thus reducing muscle contractions. BoNT-A is commonly used due to easy titration and administration (Foley et al., 2013). However, intense motor training for rehabilitation following BoNT injection has been reported to be ineffective, perhaps due to BoNT inhibiting proper transmission of the nerve impulses to the motor unit (Gandolfi et al., 2019; Rodgers et al., 2019), in effect, paralyzing the muscle. Furthermore, some patients become resistant to the effects of BoNT while others experience side effects that lessen tolerability such as allergy skin reactions, muscle weakness local pain at the site of injection, dry mouth, malaise, and transient flu-like symptoms (Coté et al., 2005; Moeini-Naghani et al., 2016).

The actions of BoNT are reversible and last for 2–4 months. In contrast, chemo denervation therapies, such as phenol and alcohol injections can cause notable scar tissue, which limits their repetitive use, moreover, if used in large dose their effect is non-reversible, as they destroy the terminal nerves.

Other forms of treatment include electrical-based stimulation techniques, such as transcutaneous electrical nerve stimulation (TENS) and functional electrical stimulation (FES), both applied as an integrative approach to physical therapy. TENS uses skin surface electrodes to apply high-frequency electrical stimulation over the spastic region. The use of TENS to treat spasticity in stroke has been shown effective as an adjunct treatment in reducing lower limb spasticity (Mahmood et al., 2019) and improving static balance and walking speed. However, a recent study that used TENS on multiple muscle groups in patients with chronic stroke and spasticity failed to find any significant reduction in spasticity when compared to sham (Pennati et al., 2021). Functional electrical stimulation (FES) uses mild electrical current to cause a muscle to contract (Okuma et al., 2003). The impulses trigger a desired function, such as contracting muscles to move a foot or lift an arm. FES can be particularly successful when applied to the peroneal nerve to assist dorsiflexion of the affected foot, because the stimulation induces activation of agonist muscles (tibialis anterior, peroneal) at the same time as relaxation of the antagonist muscles (gastrocnemius and soleus) resulting in a more normal gait pattern in spastic hemiparetic stroke survivors.

The invasive options for spasticity treatment involve surgeries to treat functional impairments resulting from spasticity. These surgeries include myelotomy, tenotomy, tendon transfers, and cordectomy to decrease muscle contraction. The abnormal sensory nerve rootlets are identified and sectioned, while the motor nerves remain intact. These techniques optimize function, in particular to improve hand opening (fingers flexor tendon) and walking (triceps surae tendon), and could prevent contractures (Thibaut et al., 2013). In general, surgical results are variable and patient-dependent (Thibaut et al., 2013).

In summary, current treatment options to improve spasticity have many adverse effects that limit their use. Neuron activation and spinal motor alterations seem to be primary components of the pathophysiology behind paresis following a brain lesion. The effects of physical therapy on spasticity are generally temporary, relatively minor, and only achieved after multiple, repetitive, task-specific therapy sessions. Drugs have side effects, and surgery has risks such as pain or infections. Therefore, there is a tremendous unmet medical need to investigate alternative treatment modalities to improve spasticity and its effects on activity in daily living.

### Neuromodulation Techniques

In recent decades, several non-invasive and painless brain stimulation techniques, including repetitive transcranial magnetic stimulation (rTMS) and transcranial direct current stimulation (tDCS), have been used to promote rapid cortical plasticity in humans. At the neuronal level, tDCS modulates the resting membrane potential in a polarity-dependent fashion: anodal stimulation increases cortical excitability in the stimulated region while cathodal stimulation decreases it (Pellicciari et al., 2013). On the other hand, rTMS modulates cortical excitability in a frequency-dependent manner, low frequencies (≤ 1 Hz) are associated with decreased cortical excitability, while higher frequencies (≤ 5 Hz) generally lead to increased cortical excitability. Although most of the studies involving tDCS and rTMS focus on the motor or cognitive recovery after stroke, few directly address the effects of these techniques on spasticity (Molero-Chamizo et al., 2021).

Trans-spinal direct current stimulation (tsDCS) is an emerging modality for non-invasive neuromodulation that induces corticospinal excitability changes and increases the motor output of multiple spinal segments in humans. It involves consistent application of a direct current over the spinal cord through a pair of electrodes (typically embedded on saline solution-soaked sponges), one placed over the targeted spinal cord segment and the other (reference) over an area distal to the spine (e.g., arm, abdomen). Direct current intensities are usually in the range of 1.5–2.5 mA, and the post-stimulation effects last from minutes to hours (Urban et al., 2010; Opheim et al., 2014).

In humans, tsDCS was pioneered in 2008 by Cogiamanian, who showed that thoracic anodal tsDCS (2.5 mA, 15 min) modulated the cervical-medullary P30 component of the posterior tibial nerve somatosensory-evoked potential for at least 20 min after the current offset. This was the first evidence that tsDCS was shown to modulate spinal cord conduction properties. Subsequent studies conducted in humans further confirmed the potential of tsDCS to modulate both ascending and descending pathways (Bocci et al., 2015; Berry et al., 2017; Sasada et al., 2017; Marangolo et al., 2020; Yamaguchi et al., 2020), as well as spinal reflexes (Priori et al., 2014).

### tsDCS Mechanisms of Action and Safety

tsDCS is a non-invasive technique considered safe in adults. No published studies have reported adverse effects during or after tsDCS, with the exception of transient redness under the electrodes. In humans, Cogiamanian et al. (2008) reported no changes in serum neuron-specific enolase, a powerful marker of neuronal damage, before and after stimulation offset.

The mechanisms of action of tsDCS have been studied in animals and subsequently confirmed that tsDCS acts on ascending/descending pathways and on segmental reflex responses, suggesting glutamatergic, GABAergic, and glycinergic system involvement and effects on spinal plasticity. Electric current can easily flow within the spinal canal through the intervertebral spaces. tsDCS-induced changes in spinal excitability occur at two key time-points, during (termed online effects) and after (termed aftereffects) current offset. The online effects on a neuron or axon depend on several features ranging from field properties (intensity, polarity, and direction) to neuroanatomical and neurophysiological properties in the targeted spinal structure (ElBasiouny and Mushahwar, 2007). Changes in membrane potentials were also associated with changes in trans-synaptic (Eccles et al., 1962) or direct α-motoneuron excitability (Hounsgaard and Kiehn, 1993). The manner by which polarizing effects change neuronal membrane excitability depends on how spinal cord fibers are spatially oriented in relation to the electric field (Terzuolo and Bullock, 1956). Hence, a given electrical field polarity can increase spinal tract excitability in the white matter and simultaneously decrease excitability in neural elements in the gray matter, or vice versa. There is some pioneering work exploring the effects of tsDCS in animals (Fuortes, 1954), showing the potential of this technique in modulating spinal circuitry (Ahmed, 2011, 2013, 2014b, 2016; Ahmed and Wieraszko, 2012; Samaddar et al., 2017).

Transmission in ascending spinal pathways has been observed with anodal tsDCS in healthy humans and tsDCS can interfere with conduction in the spinothalamic pathways. Truini et al. (2011), observed that after anodal low-thoracic and cervical stimulation, there was reduced peripheral laser-evoked potentials and improved pain tolerance, which mainly reflect peripheral Aδ fiber activation mediating pain information to the brain through the spinothalamic tract. These findings are further supported by a report of the persistent analgesic effect of tsDCS on central neuropathic pain in multiple sclerosis patients (Berra et al., 2019).

Previous studies showed that tsDCS can also modulate transmission in descending pathways. Lim and Shin (2011) reported that tsDCS (2 mA, 20 min) with the active electrode on the seventh cervical vertebra and the reference on the anterior aspect of the neck, increases corticospinal excitability assessed by motor-evoked potentials (MEP) elicited by transcranial magnetic stimulation (TMS) to the motor cortex in the upper limb, in a polarity-independent way. Additionally, Yamaguchi et al. (2020) demonstrated that tsDCS enhanced the function of the monosynaptic corticomotoneuronal pathway. They observed a shortened latency of descending motor signals and enhance voluntary motor output.

Bocci et al. (2015) reported that thoracic tsDCS-induced polarity-specific changes in corticospinal excitability last for >30 min after current offset and selectively affect responses in lower limb muscles innervated by lumbar and sacral motor neurons. This finding was supported by Sasada et al. (2017) who observed that lumbar tsDCS significantly modulated the power output of healthy volunteers during sprint cycling in a polarity-dependent way. Similarly, a trial led by Berry et al. (2017), showed that a single session of anodal tsDCS in healthy volunteers significantly improved vertical jump performance for at least 3 h, suggesting enhanced conduction of descending motor signals and persistent fatigue resistance. Other studies have shown enhanced function in the diaphragm muscle. Although only cathodal tsDCS stimulation was use, it demonstrated increased respiratory tidal volume. Taken together, these findings confirm the ability of tsDCS to modulate corticospinal excitability.

In addition to ascending and descending spinal cord pathways, tsDCS can also modulate various spinal cord circuits. In a protocol designed to investigate tsDCS effects on H-reflex homosynaptic depression, Winkler et al. (2010) showed that thoracic anodal tsDCS (2.5 mA, 15 min) induced a long-lasting decrease in homosynaptic depression and increased H-reflex, while cathodal tsDCS increased homosynaptic depression and decreased H reflex. The H_MAX_/M_MAX_ ratio, however, was unaffected by tsDCS. The lack of H_MAX_/M_MAX_ ratio modulation indicates that tsDCS had no significant influence on α-motoneuron and that its effect on homosynaptic depression arises from spinal tsDCS-induced changes at the α-motoneuron connections. Homosynaptic depression is decreased in spastic patients (Grey et al., 2008), and this reduction is correlated to the severity of spasticity in stroke patients. Therefore, this tsDCS mechanism might be a promising tool to improve spasticity.

**Figure 1:**
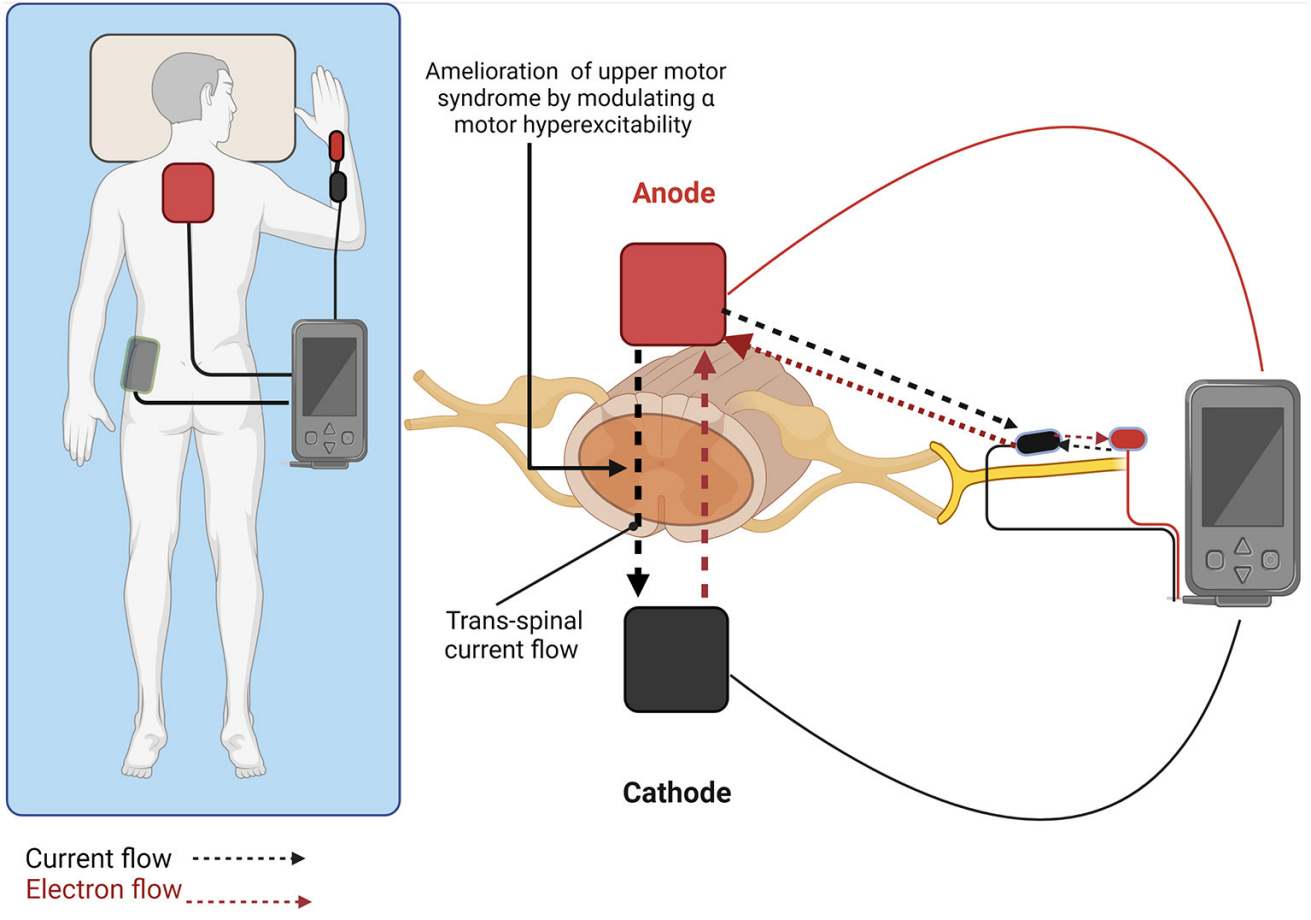
Trans-spinal direct current stimulation combined with peripheral direct current simulation (tsDCS + pDCS) promotes polarity dependent changes in muscle tone. Anodal stimulation (shown) with current flowing from the spinal cord to the periphery decreases muscle tone, while cathodal stimulation with current flowing from the periphery to the spinal cord increases muscle tone. Created with BioRender.com.

A recent elaboration of tsDCS developed for spasticity (Ahmed, 2014b) includes its combination with peripheral direct current stimulation (pDCS) to produce polarity dependent changes in muscle tone. In this configuration (Figure 1), a spinal anode is positioned along the spinal column to induce current flows from the spinal cord to the periphery to decrease muscle tone. Current flows both across the spinal cord, and down the peripheral limb. Beyond the effects of current on spinal neurons and pathways, current flow down an afflicted limb reduces the excitability of the initial segment of motor neuron axons involved in action potential initiation. Ahmed et al. showed in anesthetized mice that anodal tsDCS directly applied over the spinal cord decreased spinal excitation, while cathodal tsDCS increased spinal excitation (Ahmed, 2011). In the study, stretch-induced nerve discharges and muscle resistance during passive muscle stretches were measured in healthy mice and mice with clinical signs of spasticity following spinal cord injury (SCI). At the same time, either spinal-to-sciatic or sciatic-to-spinal tsDCS was applied (Ahmed, 2014a). This was done by utilizing tsDCS at the spinal cord level coupled with stimulation of the sciatic nerve. After anodal tsDCS, muscle resistance was reduced at all speeds tested, implying decreased spasticity. This reduction of spasticity persisted for at least 50 min after the current offset.

Conversely, both cathodal and sham tsDCS failed to reduce spasticity (Mekhael et al., 2019). While anodal tsDCS + pDCS decreased both peak muscle resistance and EMG area for up to 4 weeks after the end of the stimulation, neither cathodal nor sham had any effect (Mekhael et al., 2019).

The molecular mechanism of action of tsDCS + pDCS was also investigated. NKCC1 is a Na/K/Cl co-transporter found on spinal motor neurons and maintains intracellular chloride concentration. In mice with induced spinal cord injury, protein levels of NKCC1 were elevated following injury. Elevated NKCC1 levels result in high intracellular chloride and GABA_A_-R activation becoming excitatory instead of inhibitory. The motor neurons thus become hyperexcitable which leads to spasticity. Anodal tsDCS + pDCS was found to suppress NKCC1 protein levels, lowering intracellular chloride and enabling GABA_A_-R activation to become inhibitory and reduce spasticity. Suppression of NKCC1 activity is linked to a reduction in spasticity sustained over time, suggesting long-term changes in the excitability of spinal motor neurons through molecular level changes. These findings indicate that consecutive days of tsDCS + pDCS treatment leads to a gradual reduction and normalization of NKCC1 protein levels.

## Subsections Relevant for the Subject

We performed a literature review of clinical studies that used tsDCS as the main intervention to treat spasticity as a main or secondary outcome in different medical conditions. A summary of human studies using TsDCS to treat spasticity are summarized in Table 1.

**Table 1:** Published tsDCS clinical trials for spasticity with summary of the results.

**References**	**Intervention**	**Control**	**Condition**	**Study design**	**Outcomes**	**Results**
Ardolino et al. (2021)	tsDCS anodal 2.0 mA, 5 days delivered over the thoracic spinal cord (T10–T12)	tsDCS sham	Hereditary spastic paraplegias *N* = 11 *N* = 8 spastin/SPG4 *N* = 1atlastin1/SPG3a *N* = 1paraplegin/SPG7 *N* = 1 ZFYVE26/SPG15	Double-blind, randomized, crossover study.	- MEP - H-reflex (Hr) - F-waves - AS - Five Minutes Walking test - SPRS	- Anodal tsDCS significantly (*p* = 0.0137) decreases spasticity - Five Minutes Walking test and SPRS did not differ between the groups -H-reflex, F-waves, and MEPs unchanged over time
Paget-Blanc et al. (2019)	Trans-spinal direct current stimulation and peripheral nerve direct current stimulation (tsDCS + pDCS) five consecutive daily sessions 20 min	tsDCS sham	Patients with upper extremity spasticity following stroke *N* = 26 with upper limb hemiparesis and wrist spasticity	Single-blind crossover design study	- MTS and torque measures for Spasticity - Motor function With FMS and Wolf Motor Function Test	- tsDCS + pDCS demonstrated significant reductions (*p* < 0.05), in both MTS and torque measures on wrist flexor - FMT and Wolf Motor Function Test improved (*p* < 0.05) in the active group
Berra et al. (2019)	Anodal ts-DCS group, 10 daily 20-min sessions, 2 mA)	Sham tsDCS	Neuropathic pain in Multiple sclerosis *N* = 33 Active = 19 Control = 14	Double-blind sham-controlled trial	- NPSI - AS - FSS - In a subgroup: - NWR - NWR	- Anodal ts-DCS group showed a significant improvement in NPSI [*F*_(37, 29)_ = 5.175; *p* = 0.013] - No significant change in AS and FSS in either group
Abualait and Ibrahim (2020)	Cathodal tsDCS, and anodal tsDCS, for 20 min, with a set intensity of 2.5 mA + robot-assisted gait training 30 sessions, with 5 sessions per week for 6 weeks	Sham tsDCS	Incomplete chronic SCI *N* = 2	Pilot cross-over study Patient A received sham and cathodal tsDCS, while Patient B received sham and anodal tsDCS	−10-m walk test - MTS - Berg balance scale - Manual muscle testing - Spinal cord independence measure-III - MEP	- Sham stimulation showed no effect on spasticity - Anodal stimulation increased muscle strength - Cathodal stimulation decreased muscle strength - Sham and cathodal stimulation increase in MEP amplitudes

*MEP, Motor Evoque Potentials; AS, Asworth Scale for clinical spasticity; MTS, Modified Tardieu Scale; FMS, Fugl-Meyer Scale; NPSI, Neuropathic pain symptoms inventory; FSS, Fatigue Severity Scale; NWR, Nociceptive withdrawal reflex; TST, temporal summation threshold; SPRS, Spastic Paraplegia Rating*.

## Discussion

We have found four clinical trials utilizing tsDCS as a treatment for spasticity across different conditions including, hereditary spastic paraplegia, upper extremity spasticity following stroke, neuropathic pain in multiple sclerosis, and in incomplete chronic spinal cord injury. Three of the studies assessed changes in spasticity as the primary outcome and one of them as a secondary outcome. Three studies showed that the active tsDCS decreased spasticity and one did not.

In the first study about hereditary spastic paraplegia, the authors found improvements in spasticity and walking ability in the anodal stimulation group when compared to sham stimulation. These clinical changes persisted for up to 2 months following intervention, however, non-significant changes were observed on the neurophysiological measures in all groups, this discrepancy might be explained due to the small and heterogeneous sample, the progressive loss of corticospinal fibers, and the possibility ceiling effect due to the small number of residual fibers in this group of patients (Ardolino et al., 2021).

The second study is a first-in-human pilot study applying tsDCS + pDC in stroke patients. The authors used the paired stimulation approach to suppress spinal hyperexcitability in chronic stroke survivors to treat upper-limb spasticity. They found a significant reduction in spasticity and motor function improvement that lasted up to 5 weeks after treatment. Interestingly, patients did not receive any intervention and/or treatment during the trial duration, indicating that the observed clinical results were mostly associated to the intervention itself. The authors of this study used standardized spasticity measurements and stroke-specific, performance-based impairment index to assess improvement, thus, showing the applicability of this technique in the clinical setting (Paget-Blanc et al., 2019).

In another study on multiple sclerosis by Berra et al. (2019), the primary outcome was a change in neuropathic pain. They found that patients who received anodal tsDCS showed a significant improvement in the neuropathic pain inventory, but no significant changes were reported in spasticity, this, probably related to the and the specific features of MS patients (i.e., prevalence of secondary-progressive MS) and the complex mechanisms involved in MS spasticity including the co-occurrence of lesion at different levels (supraspinal and spinal) and with variable extension (Zaffaroni, 2010).

In the fourth study, Abualait and Ibrahim (Abualait and Ibrahim, 2020) provided cross-over tsDCS to two male patients (A and B) with SCI, both subjects received robot-assisted gait therapy during stimulation. Patient A received sham followed by cathodal tsDCS, while Patient B received sham followed by anodal tsDCS. Authors reported that motor evoked potentials amplitude increased after cathodal stimulation and deteriorated with anodal stimulation, tsDCS seemed to have a positive effect on gait parameters and spasticity in incomplete SCI. Anodal tsDCS improved lower extremity muscle strength and tone to enhance locomotor ability, while cathodal tsDCS decreased spasticity in these patients with incomplete SCI. Results must be cautiously considered. Both participants received robot-assisted gait therapy, an intervention that has profound impact on muscle functioning itself.

All these studies suggest that tsDCS can modulate motor and sensory spinal pathways depending on the electrode montages. In this literature review, tsDCS induced improvements in motor functions, spasticity and neuropathic pain.

Moreover, the approach used by Paget-Blanc et al. (2019) represented a novel intervention that combines tsDCS with pDCS for the specific treatment of muscle spasticity. This has the advantage of targeting both the spinal cord and peripheral nerves to potentiate the electrical stimulation effects to decrease spasticity.

The application of tsDCS is moving forward into different clinical conditions, there is, for instance, an ongoing larger clinical trial in upper limb post-stroke spasticity in chronic patients (more than 6 months post-stroke), and our group is preparing one study in subacute complete SCI, and another in amyotrophic lateral sclerosis (ALS).

The delivery refinement of this neuromodulation technique, such as adding stimulation channels to expand the neuromodulatory effects at different spinal segments and; and the integration with other rehabilitation techniques, such as robotic therapy, physical therapy, and occupational therapy, will enhance the efficacy of tsDCS.

In conclusion, tsDCS is a safe technique that modulates cortico-spinal excitability, with potential to be used as a treatment option in spasticity, especially when combined with other interventions to improve functional outcomes.

## Author Contributions

All authors listed have made a substantial, direct, and intellectual contribution to the work and approved it for publication.

## Funding

This research was supported by NIH-NINDS National Institute of Neurological Disorders and Stroke NINDS Cooperative Research to Enable and Advance Translational Enterprises for Devices (CREATE Devices) grant (U44 NS104138).

## Conflict of Interest

NY is employed by PathMaker Neurosystems Inc. ZA is a stockholder and consultant to PathMaker Neurosystems Inc. which manufactures the device that delivers the double stimulation. The remaining authors declare that the research was conducted in the absence of any commercial or financial relationships that could be construed as a potential conflict of interest.

## Publisher's Note

All claims expressed in this article are solely those of the authors and do not necessarily represent those of their affiliated organizations, or those of the publisher, the editors and the reviewers. Any product that may be evaluated in this article, or claim that may be made by its manufacturer, is not guaranteed or endorsed by the publisher.
